# Metastatic follicular thyroid carcinoma to the mandible: a case report

**DOI:** 10.1186/1757-1626-2-6533

**Published:** 2009-04-29

**Authors:** Sumairi Bin Ismail, Mannil Thomas Abraham, Zuraiza Binti Zaini, Hashim Bin Yaacob, Rosnah Binti Zain

**Affiliations:** 1 Department of Oral Pathology & Oral Medicine, Hospital Sultan Abdul Halim, Sungai Petani, Kedah, Malaysia; 2 Department of Oral & Maxillofacial Surgery, Hospital Tengku Ampuan Rahimah, Ministry of Health, Klang, Selangor, Malaysia; 3 Oral Cancer Research and Coordinating Centre (OCRCC), and Department of Oral Pathology, Oral Medicine and Periodontology, Faculty of Dentistry, University of Malaya, 50603 Kuala Lumpur, Malaysia

## Abstract

**Introduction:**

Metastatic lesions to the oro-facial region may be the first evidence of dissemination of an unknown tumour from its primary site.

**Case presentation:**

We described a case of metastatic follicular thyroid carcinoma to the mandible presenting with pain and loosening of teeth in a 70 years old female patient leading to extraction of the loose teeth.

**Conclusion:**

The present case emphasizes the importance of considering metastasis in the differential diagnosis of swelling related to loosening of teeth, even though the patient had no history of any malignant disease.

## Introduction

Metastatic carcinomas to oral region constitute approximately one percent of all oral malignancies. Breast, lung, adrenal, kidney, gastrointestinal tract and prostate are the most common sites for primary tumor from which metastasis occurs [1,2]. Metastatic tumours to the jaw bones are more frequently reported than those in the oral mucosa. The body of the mandible especially the premolar-molar region is the most common site of metastasis in the oral cavity [1,3]. Pain and/or paraesthesia are the most common clinical symptoms. Thyroid carcinoma metastasizes to the jaw is extremely rare accounting about 3.85% of all jaws metastases [2]. Arriving to a diagnosis is a challenge for this case as there is no previous history of a malignant disease.

## Case presentation

A 70-year-old Malay female patient reported to the outpatient department of Oral and Maxillofacial Department, Tengku Ampuan Rahimah Hospital, Klang with a complaint of pain and swelling in the left side of the angle of the mandible. She had noticed the swelling three months previously and it had increased gradually to the present size. Three months earlier she had undergone extraction of the left mandibular second premolar for complaints of mobility and pain. She also complained of bouts of nausea and vomiting for the past 1 to 2 months with loss of appetite. The medical history revealed that the patient is been treated for hypertension and diabetes with no history of malignancy. There was no history of smoking, alcohol consumption or betel quid chewing.

**Figure 1:**
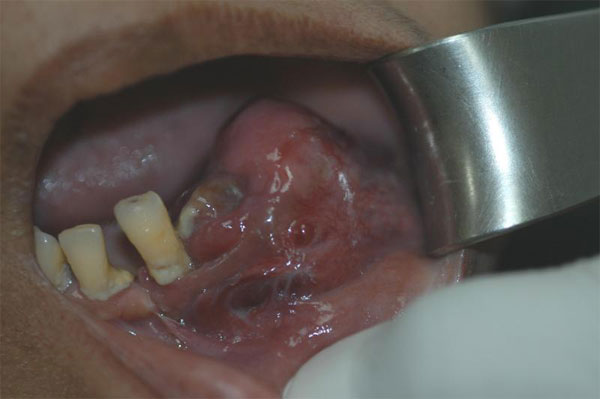
**Swelling of the left gingival area**. An intra-oral photograph showing the metastatic lesion at gingiva of the left mandibular premolar and molar area

**Figure 2:**
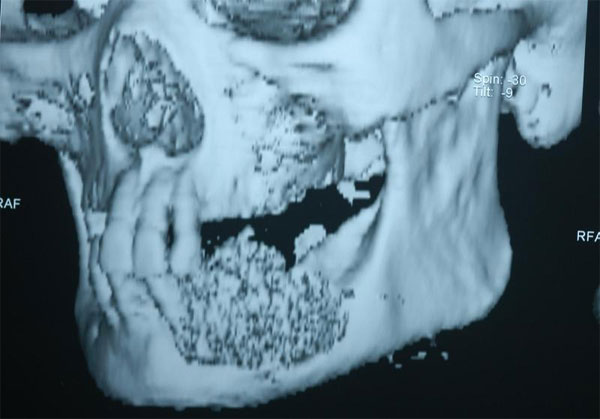
**A 3-Dimentional CT scan**. A three dimensional CT scan showing the metastatic lesion

On examination, a firm, swelling of size 3 to 4 cm was noticed in left side of the mandible, distal canine region extending to the second molar and involving the buccal sulcus at the canine region (Figure 1). On palpation, bicortical expansion of the body of the mandible was noted. Intraorally, there was an erythematous change over the region and adjacent buccal mucosa. No submandibular lymph nodes were palpable. Radiographic examination and 3-dimensional CT (computed tomography) scan showed a lesion of the left body of mandible extending from the lower left canine region to second molar region with diffuse margin leaving lower border intact (Figure 2).

**Figure 3:**
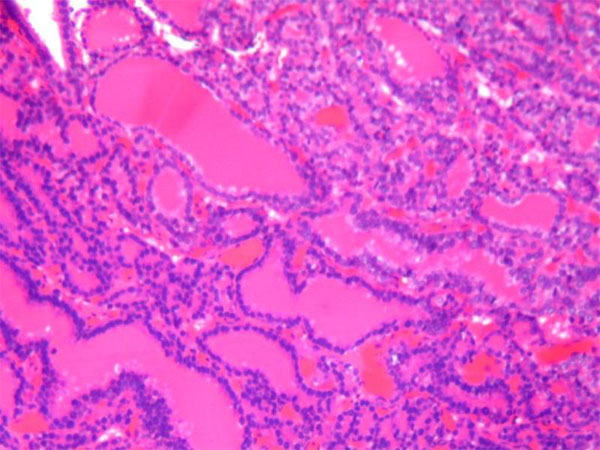
**Histopathology of The Metastatic Lesion**. A photomicrograph showing well-differentiated thyroid follicles with abundant colloid (H&E; Original magnification-20X)

An incisional biopsy was performed. The histopathological examination revealed thyroid follicles filled with colloid material (Figure 3) which is positive for PAS stain. The cells surrounding the follicles were intensely positive for thyroglobulin. The interpretation was metastatic follicular carcinoma of the thyroid.

Subsequent coronal CT scan of the neck was done showing a mass in the right thyroid region. Chest radiograph and axial CT scan were also taken which showed metastatic lesion and enlarged hilar lymph nodes.

## Discussion

Because of their rarity, metastatic tumours to the oral region are difficult to diagnose. The most common primary sites are the breast (21.8%) followed by lung (12.6%), adrenal (8.7%), kidney (7.9%), bone (7.4%), colo-rectum (6.6%) and prostate (5.6%) [2]. Most metastatic tumours to the oral region occur in patients aged 40 to 70 years [1,3].

Metastatic tumours are of great significance since some cases may represent the only symptom of an undiscovered underlying malignancy. In one third of patients, oral metastasis may be the first evidence of metastasis from its primary site [1]. In the jaw, pain, swelling, loosening of tooth and paraesthesia are the most common clinical manifestations [1,3]. Patient complaining of numb chin or mental nerve neuropathy should always raise the possibility of a metastatic disease in the mandible. A peculiar site for metastasis is the post extraction site. Hirshberg et al. reported 55 cases out of 390 cases, in which tooth extraction preceded the discovery of the metastasis [2]. The most common radiographic presentation is a radiolucent lesion with ill-defined margins. However, in approximately 5% of cases, pathological changes are not detected radiographically [2].

Follicular thyroid carcinoma (FTC) is a well-differentiated tumour which originates in follicular cells and resembles the normal microscopic pattern of the thyroid. It is the second most common cancer of the thyroid after papillary carcinoma [4]. Immunohistochemical marker for FTC is thyroglobulin, which is present in more than 95% of follicular thyroid carcinoma [5].

Distant metastases occur in 10 to 15% of patients with differentiated thyroid carcinoma [6]. Bone metastasis is the second most common site of metastasis after lung. Bone metastasis are found in 1 to 3 percent of well-differentiated thyroid carcinomas, occurring more often in follicular carcinoma and in patients more than 40 years of age [7]. Follicular thyroid carcinoma rarely gives rise to oral metastasis. The other reported sites in the oral region were parotid gland [8], tongue and labial mucosa [9].

The optimal therapy for differentiated thyroid cancer includes thyroidectomy and radiotherapy. The presence of distant metastases is associated with poor prognosis. An overall 10-year survival rate of 27% for bone metastasis of differentiated thyroid carcinoma has been reported [10]. Brennan et al. reported 40% survivors of distant follicular metastases after 5 years [11]. An early detection of metastatic disease improves the overall survival rate and treatment results.

## List of abbreviations

FTC: Follicular thyroid carcinoma; CT: Computed tomography.

## Consent

Written informed consent was obtained from the patient for publication of this case report and accompanying images. A copy of the written consent is available for review by the Editor-in-Chief of this journal.

## Competing interests

The authors declare that they have no competing interests.

## Authors' contribution

MTA analyzed and interpreted the patient clinical data regarding the clinical presentation and the radio imaging of the case. RBZ and SBI finalize the histological examination of the biopsy and SBI was the major contributor in writing the manuscript. HBY and ZBZ edited the manuscript and all authors read and approved the final manuscript.
